# Long-term prognosis of 4 children with steroid-sensitive nephrotic syndrome and relapse after 30 years of age

**DOI:** 10.1007/s13730-013-0096-8

**Published:** 2013-09-14

**Authors:** Osamu Motoyama, Ken Sakai, Kikuo Iitaka

**Affiliations:** 1 Department of Pediatrics, Toho University Medical Center, Sakura Hospital, 564-1 Shimoshizu, Sakura, Chiba 285-8741 Japan; 2 Department of Nephrology, Toho University Medical Center, Omori Hospital, Tokyo, Japan; 3 Narse Renal Clinic, Tokyo, Japan

**Keywords:** Childhood-onset nephrotic syndrome, Steroid-sensitive nephrotic syndrome, Frequently relapsing nephrotic syndrome, Prognosis

## Abstract

Some children with steroid-sensitive nephrotic syndrome (SSNS) have been reported to suffer relapses in adulthood, but the clinical course of such adults is unclear. Four children with SSNS suffered relapses after 30 years of age. Those 4 patients developed frequently relapsing nephrotic syndrome (NS) between 2 and 10 years of age. They were treated with prednisolone (PSL) combined with cyclophosphamide in 3 patients, mizoribine in 2, and cyclosporine in 1 during childhood, and with cyclosporine in 2 during adulthood. After 20 years of age, the frequency of relapses gradually decreased. The last relapse occurred between 33 and 39 years of age, and proteinuria disappeared within 1 month after the start of treatment with PSL. At the last follow-up, all 4 patients continued to receive PSL, had normal renal function, and were in complete remission of NS when they were between 33 and 41 years of age. Although the long-term outcome of SSNS is usually considered to be favorable, pediatricians should be aware that some children with SSNS may require long-term treatment during adulthood.

## Introduction

Long-term outcome of steroid-sensitive nephrotic syndrome (SSNS) is usually considered to be good. Relapses of SSNS become less frequent towards puberty, and eventually permanent remission is achieved. Recent reports have shown that almost one-third of children with SSNS suffer a relapse during adulthood [1, 2]. The clinical course in adult patients with childhood-onset SSNS is unclear.

## Case report

Four patients with childhood-onset SSNS had a relapse after 30 years of age (Table 1). Nephrotic syndrome (NS) was diagnosed in patients who had heavy proteinuria (more than 40 mg/m^2^/h) and hypoalbuminemia (serum albumin <2.5 g/dL). Patients who responded during 8 weeks of prednisolone (PSL) treatment were defined as SSNS. Relapse was defined as a reappearance of proteinuria (2+ or greater by dipstick for 3 consecutive days). Frequent relapses were defined as two or more relapses within the first 6 months after initial response or four or more relapses during any 12-month period [3]. All four patients were treated with PSL (60 mg/m^2^/day) until remission was achieved. PSL was then tapered using alternate-day doses over a six-week period. The first relapse was treated the same as the initial treatment. The PSL was tapered more slowly and the dose of PSL was determined individually according to the threshold at which the relapse occurred. Estimated glomerular filtration rate was calculated by the method of Matsuo et al. [4]. The standard deviation (SD) in patient height was calculated using the mean Japanese adult height of 170.8 ± 5.8 cm for males and 158.1 ± 5.3 cm for females.

**Table 1:** Profiles of 4 children with relapse of nephrotic syndrome after 30 years of age

	Case 1	Case 2	Case 3	Case 4
Sex	Male	Male	Female	Female
Age at onset	6 years	2 years	2 years	10 years
Number of relapses	35	53	51	44
Frequent relapser	+	+	+	+
Renal biopsy finding	FSGS	−	−	−
Treatment	PSL, CY, MZ, CyA	PSL, CY, MZ	PSL, CY, CyA	PSL, CyA
Age at last relapse	39 years	35 years	34 years	33 years
Age at last follow-up	41 years	36 years	35 years	33 years

*FSGS* focal segmental glomerulosclerosis, *CY* cyclophosphamide, *MZ* mizoribine, *CyA* cyclosporine

Their ages at the onset of NS ranged between 2 and 10 years. Neither hypertension, hematuria, nor renal failure was noted in these 4 patients. Anti-nuclear antibody was negative and serum IgA and complement levels were normal. The number of relapses of NS was between 35 and 53, and frequently relapsing NS was noted in all patients. Renal biopsy was not performed in cases 2–4. They were treated with PSL combined with cyclophosphamide (CY) in 3 patients (cases 1–3), mizoribine (MZ) in 2 (cases 1 and 2), and cyclosporine (CyA) in 3 (cases 1, 3, and 4). CY at a dose of 2 mg/kg for 8–12 weeks, MZ at a dose of 100 mg/m^2^, and CyA at a dose to target the trough level of 100 ng/mL were used. The total dose (mg/kg) of CY was 430 in case 1, 275 in case 2, and 90 in case 3. A microemulsion formulation of CyA was used in case 1. Cases 3 and 4 were treated with CyA before the microemulsion formulation was developed. Relapse was noted until 39 years old in case 1. PSL was tapered more slowly in adulthood than in childhood. During adulthood, CyA was used for 1–2 years in cases 1 and 4. After 20 years of age, the frequency of relapses gradually decreased (Table 2). At the last relapse, proteinuria disappeared promptly after the start of PSL (Table 3). Their adult heights were between −1.6 and 0.7 SD. Case 1 was obese (body mass index of 30) and was treated with antihypertensive drugs. At the last follow-up, all 4 patients continued to receive PSL, had normal renal function, and were in complete remission from NS, when they were between 33 and 41 years of age (Table 4). They were not admitted for relapses during adulthood, except for 1 admission for the 33rd relapse of case 1. Three patients (cases 1–3) were working full-time. Case 4 was a housewife with two children. Case 2 also had 2 children. Two of the patients (cases 1 and 3) were not married. There were no signs nor symptoms of insufficiency fracture, aseptic necrosis of femoral capital epiphyses, glucose intolerance, cataract, or malignant diseases in any of the 4 patients.

**Table 2:** Number of relapses per year in each decade of age

	0–9 years	10–19 years	20–29 years	30–39 years
Case 1	1.7	1.8	0.9	0.2
Case 2	3.6	1.7	0.6	0.3
Case 3	1.4	2.3	1.1	1.0
Case 4	–	2.0	2.0	1.5

**Table 3:** Dose of prednisolone and response to treatment with prednisolone at the onset of nephrotic syndrome and at last relapse

	At onset		At last relapse	
	Prednisolone (mg/m^2^)	Time to induce remission^a^	Prednisolone (mg/m^2^)	Time to induce remission^a^
Case 1	45 mg/day (46.9)	6 days	20 mg/day (9.7)	10 days
Case 2	30 mg/day (55.6)	8 days	40 mg/day (22.7)	9 days
Case 3	30 mg/day (51.7)	7 days	5 mg/day (3.6)	<4 weeks^b^
Case 4	60 mg/day (54.5)	9 days	45 mg/day (30.4)	7 days

^a^ Time from the start of treatment with prednisolone

^b^ Proteinuria was not tested until 4 weeks after treatment with prednisolone

**Table 4:** Clinical findings at last follow-up

	Case 1	Case 2	Case 3	Case 4
Height (standard deviation)	0.7	−0.8	−1.6	−1.5
Body mass index (kg/m^2^)	30	24	19	22
Blood pressure (mmHg)	132/76	130/76	90/40	120/70
Antihypertensive agents	ARB etc.	–	–	–
Serum creatinine (mg/dL)	0.7	0.7	0.6	0.5
eGFR (mL/min/1.73 m^2^)	100	99	99	113
Proteinuria	–	–	–	–
Treatment				
Prednisolone (mg/day)	2.5	15	2	10
Cyclosporine (mg/day)	200	–	–	–

*ARB* angiotensin-receptor blocker, *eGFR* estimated glomerular filtration rate

## Case 1

CY was used at the age of 7 and 10 years, and MZ between the age of 19 and 20 years. He had a 33rd relapse at the age of 29 years, and renal biopsy showed focal segmental glomerulosclerosis. His body mass index was over 30 and his renal function remained normal. Proteinuria disappeared 1 week after treatment with PSL. Focal segmental glomerulosclerosis associated with obesity was suspected [5]. An angiotensin-converting enzyme inhibitor was added after the complete remission of NS. At his 34th relapse at 34 years old, he went into complete remission within 2 weeks after the treatment with PSL, and PSL was tapered for 3 years. At his 35th relapse at 39 years old, CyA was started, and angiotensin-converting enzyme inhibitor was changed to angiotensin-receptor blocker and calcium channel blocker.

## Case 2

He was treated with CY at 8 and 15 years of age and MZ for 1 year at 13 years of age. He had 6 relapses between 20 and 29 years of age. He developed his 52nd relapse at 31 years of age and was treated with PSL for 8 months. His daughter also developed SSNS [6]. At 35 years of age, his 53rd relapse occurred, and PSL was on a tapering dose at the last follow-up.

## Case 3

CY, followed by CyA for 1 year, was used for growth impairment due to continuous treatment with PSL at 5–10 mg/day at 13 years of age [7]. Her adult height reached −1.6 SD. After 30 years of age, 6 relapses occurred when PSL was tapered to 1 mg/day. At her 51st relapse in 34 years old, she was treated with PSL 5 mg/day, and her proteinuria disappeared within 1 month. She was on PSL 2 mg/day at the last follow-up.

## Case 4

CyA was used between 24 and 25 years of age. She had 4 relapses during treatment with CyA, and CyA was discontinued at her first pregnancy. She had relapses during pregnancy at 26 and 31 years of age and received PSL treatment. She delivered a normal baby in 2 pregnancies [8]. After her second delivery, PSL was withdrawn. Her 42nd to 44th relapses occurred between 32 and 33 years of age, and PSL treatment was restarted.

## Discussion

Relapses of SSNS become less frequent towards puberty, and eventually permanent remission is achieved. Studies from the 1980s reported that no more than 10 % of children had additional relapses in adulthood. More recent reports indicated a relapse rate after 18 years of age of between 27 and 42 % [1, 2]. Most patients who have relapsed during adulthood developed NS at a young age and were frequent relapsers during childhood [2, 9, 10]. Between 1972 and 2013, 148 patients were diagnosed with SSNS before 15 years of age at Toho University Omori Hospital and Sakura Hospital. Of the 34 patients who were followed after 20 years of age, 12 had at least one relapse after 20 years. The mean onset age for the 12 patients was 6.8 ± 3.6 (2.2–12.7) years, and 11 of them were frequent relapsers. Of the 5 patients who relapsed after 20 years of age and were followed after 30 years of age, 4 patients had one or more relapses after 30 years. To our knowledge, only a few patients who relapsed after 30 years of age have been reported. Relapses in a 33-year-old patient and a 39-year-old patient were reported [9, 11]. Kyrieleis et al. [12] reported 5 patients whose onsets of frequently relapsing minimal change nephrotic syndrome (MCNS) ranged between 1 and 3 years of age, and who had relapses between 32 and 42 years of age. However, the clinical courses of these patients in adulthood were not described.

Patients with adult-onset MCNS who have transient hypertension and impaired renal function during the nephrotic phase are not rare, and therapeutic response is slower than in children. According to a report by the International Study of Kidney Disease in Children, 85–90 % of children with NS achieved complete remission within 4 weeks and 90–95 % within 8 weeks after the start of steroid treatment. In adult patients with MCNS, response to steroid treatment may take up to 15 weeks. In some studies of adult patients given PSL, remission occurred in 50–60 % of patients after 8 weeks of treatment, and in 70–75 % after 16 weeks [13]. In the 4 patients presented here, the time period from the start of treatment with PSL to the induction of complete remission was similar to that of childhood NS. Frequency of relapse decreased with age, and renal function was normal after 30 years of age. The prognosis was similar to the children with SSNS, despite their prolonged course of NS. There does not seem to be any proven way to predict the individual relapse courses of patients with SSNS at onset [1]. Evaluating the number of relapses during a long-term course, as shown in Table 2 of this report, may predict the frequency of relapse during adulthood in some patients.

Both increased duration and a higher dose of PSL treatment leads to prolonged remission. The Cochrane database of systematic reviews suggested that duration of PSL therapy was more important than the dose of PSL. In children with SSNS treated with PSL for 7 months, frequency of relapse over the course of 2 years significantly decreased compared with that for SSNS children treated with PSL for 2 months [14]. At relapse in adult patients, PSL should be continued for 7 months.

Case 1 presented here suffered a relapse at 39 years of age, and a relapse in a 42-year-old patient has also been reported [12]. Although the long-term outcome of SSNS is usually considered to be favorable, the chronic course and prolonged treatments involved affect the quality of life of children and also that of their families. Prolonged steroid treatment may cause short stature, obesity, and hypertension in adulthood [2]. Social performance and quality of life seemed to be relatively good in the 4 patients reported here. Pediatricians should be aware that some children with SSNS may require long-term treatment even after they have entered adulthood.
